# Association Between Multimorbidity and Root Caries Among Older American Adults

**DOI:** 10.3390/dj13060232

**Published:** 2025-05-23

**Authors:** Saif Alyamani, Rolla Mira, Wael Sabbah

**Affiliations:** Faculty of Dentistry, Oral and Craniofacial Research, King’s College London, London SE5 8AF, UK; saif_khalid_s.alyamani@kcl.ac.uk (S.A.); rolla.mira@kcl.ac.uk (R.M.)

**Keywords:** root caries, multimorbidity, older adults, oral health, smoking, socioeconomic status

## Abstract

**Objectives:** The objective of this study was to examine the relationship between multimorbidity and root caries among older American adults. **Methods:** Data from the National Health and Nutrition Examination Survey (NHANES) 2017–2020, a cross-sectional nationally representative survey of civilian noninstitutionalised Americans was used. The analysis included dentate adults aged 50 years and older. The presence of root caries or root restoration was assessed through clinical dental examinations. Multimorbidity was identified by the existence of five common and serious chronic diseases, namely, diabetes, cancer, coronary heart disease, chronic pulmonary disease and stroke. An aggregate variable of these five conditions was created. Logistic regression models were used to assess the association between multimorbidity and root caries (outcome), adjusting for sociodemographic factors and health behaviours. **Results:** The analysis included 3274 dentate participants (mean age: 63.6 years), 18.2% had root caries, while 14%. had multimorbidity (two or more conditions). The mean number of multimorbidities was 0.65. The count of multimorbidity was significantly associated with root caries, with odds ratios (OR) of 1.26 (95% Confidence Interval (CI): 1.03, 1.52) in a model adjusting for age, gender, ethnicity, income, and education. After additionally adjusting for dental visits and smoking, the association between multimorbidity and root caries lost statistical significance (OR 1.20, 95% CI: 0.97, 1.48). **Conclusions**: Individuals with greater numbers of chronic conditions appeared to have higher probabilities of having root caries. However, the significant association was eliminated after accounting for strong behavioural predictors of root caries. The higher level of root caries among those with multimorbidity could be attributed to xerostomia caused by being on multiple medications. The findings highlight the need for interventions to prevent root caries among those with multimorbidity.

## 1. Introduction

Multimorbidity, defined as “the coexistence of two or more chronic diseases in an individual”, significantly impacts health outcomes, healthcare utilisation, and quality of life among older adults [1]. The prevalence of multimorbidity has been increasing in the United States, driven by the aging population and lifestyle factors such as sedentary behaviour and poor diet. According to the Centres for Disease Control and Prevention (CDC), 4 out of 10 American adults live with multiple chronic conditions [2].

Root caries, prevalent among older adults, significantly impacts oral health and quality of life. It leads to tooth surface loss and pain, which affects eating and nutritional intake [3]. Oral diseases also detrimentally affect psychological and social wellbeing, reducing self-esteem and social interaction and imposing substantial financial burdens on individuals and healthcare systems. Thus, effective prevention and management of root caries are essential to mitigate their negative consequences [4].

Oral health, particularly root caries, is a significant yet often overlooked condition among older adults with multimorbidity. Root caries, characterised by decay on the tooth’s root surface, typically occurs when the root becomes exposed due to gingival recession [5,6,7]. Factors contributing to root caries include poor oral hygiene, reduced salivary flow (often a side effect of polypharmacy), and dietary habits [8,9]. Periodontal disease, a key factor in root caries and tooth loss, is associated with various systemic disorders due to inflammation [10,11]. Periodontitis often involves gingival recession and exposing root surfaces to oral bacteria. Compared with the enamel on crown surfaces, root surfaces are more susceptible to dental caries [12]. Despite advancements in dental care, root caries remains a prevalent issue, particularly among those with compromised health due to multiple chronic conditions.

Furthermore, individuals with multimorbidity are usually on multiple medications, which can lead to tooth loss and medication-induced salivary gland dysfunction (MISGD). According to Villa et al. [13], it is important to note that anticholinergics, opioids, antidepressants, bronchodilators, and certain cardiovascular drugs can cause a dry mouth. Chronic xerostomia increases susceptibility to dental caries and oral infections, as individuals with multimorbidity and multiple medications may experience higher rates of tooth loss [14,15]. Targeting prevention for those experiencing medication-induced xerostomia can help prevent oral disease.

The interaction of oral and systemic health is well documented, with conditions such as diabetes, cardiovascular disease, and respiratory diseases showing bidirectional relationships with periodontal diseases [16,17,18]. Chronic hyperglycaemia can lead to decreased saliva production as a result of nerve damage, worsen periodontal disease, and increase the likelihood of root caries in individuals with poorly controlled diabetes [19,20]. However, the relationship between multimorbidity and root caries among older adults has not been sufficiently examined. Understanding this relationship is essential for developing successful strategies to enhance oral health and wellbeing in older adults. Given the increasing prevalence of multimorbidity and its associated healthcare challenges, recognising its impact on oral health can lead to better management strategies and improved health outcomes. While a few studies have examined the relationship between multimorbidity and tooth loss [21,22,23], to the best of our knowledge, no studies have examined the association between multimorbidity and root caries. The objective of this study was to examine the association between multimorbidity and root caries among older adults using nationally representative survey of the US population. The hypothesis of this study is that older adults with multimorbidity will have higher chances of root caries.

## 2. Methods

The study utilised data from the National Health and Nutrition Examination Survey (NHANES), 2017–2020, a cross-sectional nationally representative survey of civilian noninstitutionalised Americans. NHANES includes a comprehensive assessment of the participants’ health status, including detailed medical histories, dental examinations, behaviours, and use of dental services. Dental health was evaluated by licensed dentists who assessed the presence of root caries through clinical examinations. This study included dentate adults aged 50 years and older who underwent dental examinations and had complete data on the selected conditions and other variables included in the analysis. Given that the data are publicly available, ethical approval was not required for this analysis

### 2.1. Outcome Variable

The presence of root caries and/or restorations was recorded as a binary variable (yes/no). Root caries assessment targets teeth with gingival recession, identified by colour, texture, and tactile response. Status was recorded as “root caries detected”, “no root caries”, “cannot be assessed”, and “missing”, then recoded to exclude missing values and to record “0” for no root caries and “1” for root caries; the same process was applied for root caries restorations. Both variables were combined to create an aggregate “root caries experience” variable, which was used in the analysis.

### 2.2. Main Exposure

Multimorbidity is the main exposure and was indicated by self-reported diagnosis of five chronic conditions. These included five of the most common and serious chronic diseases, namely, diabetes, cancer, coronary heart disease, chronic pulmonary disease, and stroke. These conditions were selected because they are the most common and leading causes of disability and mortality among older Americans [24]. As they are life-threatening conditions, they are all linked to several medications that are essential for the participants’ life, as opposed to other conditions. Furthermore, they are associated with heightened systemic inflammation, which has a common link to periodontal disease [25,26,27,28]. It is worth noting that conditions such as dementia and Alzheimer disease could not be included, as people with these conditions were not included in the survey. Each condition was recorded as a binary variable (yes or no), and each participant’s total number of conditions was summed to create an aggregate variable of multimorbidity, a count variable ranging from 0 to 5. This count variable was used in the analysis. An additional categorical variable indicating whether a participant had multimorbidity (2 conditions or more) was also created.

### 2.3. Covariates

These included demographic variables such as age in years, sex, and race/ethnicity, which was recoded as White, Black, Hispanic, and other ethnicities. Socioeconomic factors included education, which was recoded into three groups: less than high school, completed high school, and university graduate. Income was indicated by the income-to-poverty ratio, which is the ratio between household income and the poverty threshold, and it was used as a continuous variable. Behavioural factors included smoking status, which was categorised as current smokers, former smokers, or never smokers (smoked fewer than 100 cigarettes in their life). Dental visits were categorised to indicate a visit within two years versus less often or never. The availability of any medical insurance was also included in the analysis.

### 2.4. Statistical Analysis

A description of all the variables included in the analysis was assessed to outline the characteristics of the study population. The distribution of root caries experience within each variable was also assessed, and within each included chronic condition. The prevalence of multimorbidity was also assessed within all variables included in the analysis.

Two logistic regression models were constructed to assess the relationship between multimorbidity and root caries experience. The first model was adjusted for age, sex, ethnicity, income, and education. The second model was additionally adjusted for smoking, dental visits, and health insurance. All analyses were conducted using STATA’s survey (version 18) command to account for the complex survey design and the weights of the NHANES data.

## 3. Results

After exclusion of those under 50 years and those who did not have assessments of root caries, the study included 3274 older adults aged 50 years or over, with a mean age of 63.6 years (95% Confidence Interval (CI): 62.9, 64.1). There was no difference in demographic factors between those over 50 who were included and excluded from the study. Table 1 shows the characteristics of the study population and the distribution of root caries experience within each variable. The prevalence of root caries experience was 18.2 (95% CI: 14.5, 22.5). Mean multimorbidity was 0.64 (0.60, 0.69). The prevalence of multimorbidity (two or more conditions) was 14.0% (95% CI: 12.2, 16.1). The mean of multimorbidity was significantly higher among those with root caries (0.84, 95% CI: 0.68, 0.99) than those without root caries (0.60, 95% CI: 0.55, 0.65) (Table 1). The prevalence of root caries was higher among men than women, among Black Americans than White Americans, among those with lower education than those with higher education, and among current smokers and those with no health insurance (Table 1).

The prevalence of root caries was 28.3 among those with multimorbidity (two or more conditions) and 16.5 among those with no multimorbidity. Furthermore, the prevalence of root caries was higher among individuals with each of the included chronic conditions. Root caries with higher among those with diabetes (22.7) than those without diabetes (17.1), among those with coronary heart disease (22.8) than those without (17.7), among those with those with cancer (20.8) than those without (17.5), among those with stroke (23.2) than those without (17.9), and among those with chronic pulmonary disease (30.5) than those without (16.5).

Table 2 shows the prevalence of multimorbidity (two or more conditions) within all variables included in the analysis. Multimorbidity was higher among men (17.4) than women (11.1), among current smokers (21.9) than never smokers (10.1), and among those with fewer dental visits (17.6) than those with more visits (13.1). Multimorbidity was also higher among those with root caries than those without (Table 2).

Table 3 shows the results from the logistic regression models for the association between multimorbidity and root caries. In Model 1, adjusting for demographic and socioeconomic factors, multimorbidity was significantly associated with root caries. For each additional chronic condition, there was a higher odds ratio (OR) 1.26 (95%CI: 1.03, 1.52) for having root caries. After additionally adjusting for strong behavioural predictors of root caries, namely smoking and dental visits, the association between multimorbidity and root caries lost statistical significance but remained in the same direction. Smoking appeared to be the strongest predictor of root caries, with current smokers having higher odds for root caries than never smokers (OR: 3.05, 95%CI: 2.05, 4.53) (Table 3). Higher income, having medical insurance, and dental visits showed significantly lower odds for root caries.

## 4. Discussion

This study examined the relationship between multimorbidity and root caries among older American adults. The risk of root caries was higher among participants with a greater number of chronic conditions. However, after accounting for strong behavioural predictors of root caries such as smoking and dental visits, the association between multimorbidity and root caries lost statistical significance but remained in the same direction. The results, to some extent, support the hypothesis that individuals with multimorbidity are vulnerable to root caries.

Earlier studies found that multiple chronic conditions were associated with tooth loss [21,22,23]. However, there is a lack of studies that have assessed the relationship examined in the current analysis between multimorbidity and root caries. Nevertheless, recent research highlighted that individuals with multimorbidity, who often take multiple medications, face increased risks of the subjective sensation of a dry mouth (xerostomia) and hyposalivation caused by drugs such as anticholinergics, bronchodilators, and cardiovascular medications. Xerostomia induced by these medications impacts oral health, leading to higher rates of periodontal disease, dental caries, and tooth loss, especially among older adults [7,21]. On the other hand, individual chronic conditions were found to be associated with root caries, for example, diabetes, cognitive decline and Alzheimer’s disease and cardiovascular diseases. The relationship between these specific conditions and root caries is often attributed to a decline in self-care and subsequently in oral hygiene with conditions such as cognitive decline, Alzheimer’s disease, and stroke [29]; medication-induced xerostomia [14]; the impact on periodontal conditions of diabetes [28], leading to root exposure [30]; and the effect of common risk factors such as smoking [31].

It is worth noting that prevalence of root caries experience was higher among individuals with each of the included five conditions. Gingival recession and periodontal diseases are the proximal risk factor for root caries. Several chronic conditions are linked to periodontal diseases, including the conditions assessed here [10,11,26]. Among diabetic patients, poor glycaemic control is linked to cariogenic bacteria [32] and affect salivary flow [33]. Bidirectional associations were also found for cardiovascular disease, diabetes, and chronic pulmonary disease with periodontal diseases [16,17,18]. Inflammatory markers for coronary heart disease and stroke are found among patients with periodontal diseases [34], which lead to root exposure and a higher risk for root caries. Chronic pulmonary diseases are linked to greater accumulation of plaque and poor oral hygiene [35]. Furthermore, they are more likely to use inhaled medications, which are linked to a dry mouth, which impacts dental caries. Use of inhaled medications is also linked to a tendency to consume sweetened food [35]. Treatment for cancer often includes radiation therapy, which impacts dental tissues, making them vulnerable to caries [36], in addition to the impact of the multiple medications used for cancer treatment on salivary flow.

It is also possible that the association between multimorbidity and root caries observed here could be attributed to the effect of adverse socioeconomic conditions on both multiple chronic conditions [24] and root caries [24,37], and common risk factors such as smoking and other health risk behaviours.

In the current analysis, the association between multimorbidity and root caries was independent from socioeconomic factors and ethnicity. However, behavioural factors, particularly smoking, eliminated the statistically significant association. This finding underscores the detrimental impact of smoking on oral health. Smoking can reduce salivary flow, increase dental biofilm formation, and cause direct damage to oral tissues, all of which contribute to the development of root caries [38,39]. Furthermore, the increased risk of root caries among former smokers observed here suggests that the harmful effects of smoking may persist even after smoking cessation, indicating long-lasting effects, potentially making it more challenging to maintain oral health even after quitting [40]. Lack of dental visits was another important predictor in the current analysis, which was shown to be linked to both root caries and financial abilities [37]. It is worth highlighting that women were at a higher risk of root caries than men, which could be attributed to the inclusion of more older women who maintained their teeth [29]. While education did not show a significant association with root caries, higher income was associated with lower odds, which is consistent with the literature demonstrating socioeconomic inequalities in root caries [28]. On the other hand, while the prevalence of root caries was higher among Black than White American, there was no significant difference in the fully adjusted model.

The study’s findings highlight the need for comprehensive public health strategies that address both behavioural and socioeconomic determinants of health. Smoking cessation programmes, improved access to dental care, and policies to reduce socioeconomic inequalities are crucial for improving oral health outcomes in older adults. These strategies should be integrated into broader health promotion efforts to ensure that oral health is not neglected in the context of overall health management.

The strengths of this study are in examining the relationship between multimorbidity and root caries, which has not been tested before, using a nationally representative survey and adjusting for several socioeconomic and behavioural factors. There are a few limitations worth mentioning. The cross-sectional data do not support conclusions about causality or temporality. The use of self-reported diagnoses of medical conditions and self-reported data for behaviours and socioeconomic factors is subject to recall bias. While the analysis included the most common and most serious chronic conditions in the American population, other conditions such as arthritis, depression, and dementia were not included. The inclusion of additional conditions could have affected the findings of the study. Other factors that could affect root caries such as oral hygiene practices were not available in the survey. Future studies with longitudinal designs could provide more definitive insights into the temporal relationships and mechanisms underlying the observed associations.

## 5. Conclusions

This study demonstrated that root caries was more likely to occur among older adults with multimorbidity. The study also highlighted the determinantal role of some risk factors, such as smoking, that affect multimorbidity and root caries. The findings emphasise the importance of incorporating oral health into chronic disease management to enhance older adults’ wellbeing and quality of life.

## Figures and Tables

**Table 1:** Characteristics of the study population and distribution of root caries experience among older Americans—NHANES 2017–2020 (N = 3274).

Variables		Total Percentage (95% CI)	Percentage/Mean with Root Caries (95% CI)	*p* Value *
Sex	Male	45.7 (43.66, 47.76)	21.1 (16.8, 26.1)	<0.01
	Female	54.3 (54.24, 56.34)	15.7 (12.1, 20.1)	
Age (mean)	Without root caries	63.6 (62.9, 64.3)	63.4 (62.6, 64.1)	<0.05
	With root caries		64.6 (63.5, 65.7)	
Race/ethnicity	Hispanic	10.6 (8.5, 13.2)	16.9 (11.3, 24.3)	<0.05
	Whites	71.8 (66.6, 76.5)	16.9 (13.1, 21.5)	
	Black	9.3 (6.8, 12.6)	26.9 (20.8, 34.1)	
	Other races	8.2 (6.3, 10.7)	20.9 (13.9, 30.4)	
Education	<High school	9.3 (8.2, 10.6)	27.7 (19.5, 37.7)	<0.001
	High school	28.3 (25.2, 31.7)	22.0 (17.6, 27.1)	
	College	62.4 (59.4, 65.3)	15.0 (11.8, 18.9)	
Income to poverty ratio (mean)	With root caries	3.4 (3.3, 3.5)	3.6 (3.4, 3.7)	<0.001
	Without root caries		2.7 (2.5, 2.9)	
Medical insurance coverage	Yes	93.5 (91.9, 94.8)	18.91 (15.28, 23.16)	<0.001
	No	6.5 (5.2, 8.1)	34.6 (24.4, 46.5)	
Smoking status	Never smoked	57.0 (53.3, 60.7)	12.8 (9.6, 16.8)	<0.001
	Former smoker	31.8 (29.1, 34.7)	20.4 (15.1, 26.9)	
	Current smoker	11.1 (9.1, 13.6)	39.7 (29.6, 50.6)	
Last dental visit	Never or more than 2 years	19.7 (17.3, 22.4)	32.1 (23.7, 31.7)	<0.001
	More often	80.3 (77.6, 82.7)	14.9 (11.5, 19.3)	
Multimorbidity (Mean)	With root caries	0.64 (0.60, 0.69)	0.84 (0.68, 0.99)	<0.01
	Without root caries		0.60 (0.55, 0.65)	

* p value from Chi-Square or *t*-test.

**Table 2:** Prevalence of multimorbidity (two or more conditions) within all variables included in the analysis, among older Americans—NHANES 2017–2020 (N = 3274).

Variables		Percentage/Mean with Root Caries (95% CI)	*p* Value *
Sex	Male	17.4 (14.7, 20.5)	<0.01
	Female	11.1 (8.9, 13.6)	
Age (mean)	No multimorbidity	62.8 (62.1, 63.5)	<0.001
	Multimorbidity	68.7 (67.4, 70.1)	
Race/ethnicity	Hispanic	8.7 (6.9, 11.1)	<0.001
	Whites	15.3 (13.0, 17.9)	
	Black	11.7 (9.6, 14.2)	
	Other races	12.2 (9.1, 16.1)	
Education	<High school	14.9 (11.3, 19.4)	<0.05
	High school	17.5 (13.7, 22.1)	
	College	12.3 (10.2, 14.8)	
Income to poverty ratio (mean)	No multimorbidity	3.5 (3.3, 3.6)	Insignificant
	Multimorbidity	3.1 (2.8, 3.4)	
Medical insurance coverage	Yes	14.4 (12.5, 16.6)	Insignificant
	No	8.2 (3.8, 16.8)	
Smoking status	Never smoked	10.1 (8.3, 12.3)	<0.001
	Former smoker	18.3 (14.2, 23.2)	
	Current smoker	21.9 (15.4, 30.1)	
Last dental visit	Never or more than 2 years	17.6 (14.3, 21.6)	<0.05
	More often	13.1 (11.3, 15.3)	
Root caries experience	Yes	21.8 (15.4, 30.0)	<0.01
	No	12.3 (10.5, 14.3)	

* p value from Chi-Square or *t*-test.

**Table 3:** Logistic regression analysis showing associations between multimorbidity and root caries experience among older American adults—NHANES, 2017–2020 (N = 3274).

		Model 1	Model 2
		Odds Ratio (95% Confidence Intervals)	
Sex (Reference: males)		0.65 ** (0.50, 0.83)	0.76 * (0.59, 0.97)
Age (years)		1.01 (0.50, 1.03)	1.02 * (1.01, 1.04)
Race/ethnicity (reference: White)	Black	1.36 (0.88, 2.10)	1.30 (0.84, 2.01)
	Hispanic	0.70 (0.41, 1.22)	0.81 (0.47, 1.39)
	Other ethnicities	1.12 (0.72, 1.74)	1.18 (0.75, 1.85)
Education (reference: <high school)	High school	0.89 (0.60, 1.31)	0.95 (0.62, 1.45)
	University degree	0.76 (0.52, 1.12)	0.86 (0.47, 1.28)
Income to poverty ratio (higher income)		0.72 *** (0.66, 0.79)	0.80 *** (0.73, 0.88)
Multimorbidity		1.26 * (1.03, 1.52)	1.20 (0.97, 1.47)
Smoking (reference: never smoked)	Former smoker		1.53 * (1.02, 2.38)
	Current smoker		3.05 *** (2.05, 4.53)
Medical insurance (reference: no insurance)			0.54 ** (0.36, 0.81)
Dental visits (reference: more than 2 years)			0.64 * (0.42, 0.96)

Model 1 is adjusted for sex, age, race/ethnicity, education, income, and multimorbidity. Model 2 is additionally adjusted for smoking, medical insurance, and dental visits. * *p* < 0.05, ** *p* < 0.01, *** *p* < 0.001.

## Data Availability

Data used in this research is available on https://wwwn.cdc.gov/nchs/nhanes/continuousnhanes/default.aspx?Cycle=2017-2020 (accessed on 25 March 2025).
